# Negative life events and the risk of depression: Findings from Indonesia Family Life Survey 2014/2015

**DOI:** 10.1371/journal.pone.0319137

**Published:** 2026-01-09

**Authors:** Irmansyah Irmansyah, Ida Ayu Mas Amelia Kusumaningtyas, Aryana Satrya, Laura Anselmi, Jonathan Gibson, Jack Wilkinson, Sri Idaiani, Budi Anna Keliat, Dwidjo Susilo, Hasbullah Thabrany, Herni Susanti, Helen Brooks, Penny Bee, Matthew Sutton, Asri Maharani

**Affiliations:** 1 Research Centre for Public Health and Nutrition, National Research and Innovation Agency, Cibinong, Indonesia; 2 Global Health Research Group on Sustainable Treatment for Anxiety and Depression in Indonesia, Universitas Indonesia, Depok, Indonesia; 3 Department of Management, Universitas Indonesia, Depok, Indonesia; 4 Division of Population Health, Health Services Research & Primary Care, University of Manchester, Manchester, United Kingdom; 5 Research Centre for Preclinical and Clinical Medicine, National Research and Innovation Agency, Jakarta, Indonesia; 6 Department of Nursing, Universitas Indonesia, Depok, Indonesia; 7 Department of Public Policy and Management, Universitas Gadjah Mada, Yogyakarta, Indonesia; 8 Division of Nursing, Midwifery & Social Work, University of Manchester, Manchester, United Kingdom; Maulana Azad Medical College, INDIA

## Abstract

**Background:**

Negative life experiences are well-established risk factors for mental health problems, yet evidence from low- and middle-income countries remains limited. Many studies also overlook area-level factors that may influence these relationships. This study aimed to examine the association between negative life events and depression among individuals in Indonesia, accounting for both individual and area-level characteristics.

**Methods:**

This cross-sectional study used data from 31,446 individuals aged 15 years and older who participated in the Indonesia Family Life Survey Wave 5 (IFLS-5), conducted in 2014–2015. Depression was assessed using the 10-item Center for Epidemiologic Studies Depression Scale (CES-D-10), a validated instrument measuring depressive symptoms during the past week. Negative life events, including chronic illness (self or family member), natural disasters or accidents, and deaths of family members within the past year, were evaluated. Multilevel logistic regressions accounted for the hierarchical data structure and adjusted for demographic, socioeconomic, behavioural, and district-level characteristics.

**Results:**

The prevalence of depression was 23%. Experiencing one negative life event increased the odds of depression (adjusted odds ratio [AOR] = 1.22, 95% confidence interval [CI]: 1.15–1.30), while two or more events further elevated the odds (AOR = 1.55, 95% CI: 1.32–1.83), adjusted for covariates. Individually, chronic illness (AOR = 1.25, 95% CI: 1.17–1.33), natural disasters or accidents (AOR = 1.41, 95% CI: 1.15–1.73), and deaths of family members (AOR = 1.14, 95% CI: 1.03–1.26) were significantly associated with depression.

**Conclusion:**

Multiple and specific negative life events substantially increase the risk of depression among Indonesians. These findings highlight the importance of integrating culturally sensitive mental health interventions within community and healthcare settings.

## Background

The global burden of depression is profound, affecting approximately 280 million people, or about 3.8% of the world’s population in 2019 [1]. Recognised as a leading cause of disability, depression significantly diminishes the quality of life and imposes a substantial economic burden through lost productivity and healthcare costs [2]. Depression caused 46.86 million (95% uncertainty interval [UI] 32.93–63.80) disability-adjusted life years (DALYs) worldwide in 2019, which equates to an age-standardised DALY rate of 577.75 (405.79–788.88) per 100,000 [3]. The COVID-19 pandemic has further intensified the global mental health crisis, with studies indicating that the prevalence of pandemic-related depression ranges from 14.3% to 24.3% [4]. Characterised by low mood and a range of emotional, cognitive, physical, and behavioural symptoms [5], depression is associated with chronic illnesses [6], increased risk of suicidal ideation [7,8], and higher mortality rates [9,10]. Furthermore, depression can significantly decrease quality of life and lead to disability [11,12]. Understanding the factors contributing to depression in specific populations is crucial for developing effective preventive interventions.

Studies consistently link negative life events to increased risk of mental health problems, including depression [13–16]. These events can encompass experiences of the death of someone close, serious health events or illness, or challenging socioeconomic circumstances, all of which can have lasting negative consequences [17]. Furthermore, critical life events perceived as unwanted, uncontrollable, or life-threatening can significantly impact mental well-being and self-rated health, increasing the risk of depression, anxiety, and even post-traumatic stress disorder [18].

Several studies support the connection [19–22]**.** A South African study linked serious illness/death in family members to depression [19], aligning with research highlighting specific negative events’ cumulative impact on older adults’ depression [20]. A proposed model suggests that reducing exposure to negative life events could alleviate depression and vice versa [21]. Another study adds a cognitive dimension, finding that negative life events interact with cognition to influence depression in young adults [22]. These studies emphasise the importance of considering negative life events when understanding and addressing depression.

Some limitations are evident in the emerging literature. Most studies have been conducted in high-income countries, including the UK [13], the Netherlands [23], Hong Kong [24] and the US [25]. Low and middle-income countries (LMICs) face a rising burden of mental illness, with depression projected as the third-largest source of disease burden by 2030 [26–28]. Studies in LMICs, including China and Nigeria, are limited and have focused on specific populations, such as older adults [29] or women living in slum areas [17]. Studies in LMICs are essential to understanding how cultural, economic, and social contexts influence the relationship between negative life events and depression. The unique stressors faced by populations in LMICs, such as poverty, political instability, and limited access to mental health resources, may alter the dynamics of this relationship.

Furthermore, none of the studies have adequately incorporated area-level variables that may influence the relationship between negative life events and depression. Factors such as access to healthcare, area deprivation, and local cultural norms can significantly impact how individuals experience and cope with negative life events. Research indicates that individuals with better access to healthcare resources are more likely to seek help and receive appropriate interventions [30], which can buffer against the psychological consequences of negative life events. To address these gaps, we explore the associations using large household and area-level datasets from Indonesia.

Indonesia, a middle-income country [31] with a large and diverse population, ranks among the top two nations in the Southeast region with the largest mental health burden. The GBD study shows a rise in depression prevalence in the country from 19 million (1990) to 27 million (2019), with DALYs due to depression also increasing from 303/100,000 population to 369/100,000 population [1].

Researching negative life events in Indonesia is particularly valuable due to the country’s frequent exposure to a wide range of adverse events, including natural disasters. As an archipelagic nation in the Pacific Ring of Fire, it is particularly susceptible to various natural calamities, including earthquakes, tsunamis, and volcanic eruptions [32]. These events can lead to trauma, displacement, loss, and increased psychological distress among affected individuals, contributing to mental health issues. Studies show increased depression and PTSD after disasters globally [33–35]. Studies in Indonesia confirm this. A study in Aceh and Nias provinces following a huge tsunami disaster in 2004 showed that a significant number of Aceh and Nias residents experienced psychological symptoms with internally displaced persons (IDPs), experiencing more severe effects than non-IDPs [36]. Economic losses resulting directly from the disaster amplified the risk of depression following the Jogjakarta Earthquake in 2016 [37].

Furthermore, Indonesia’s unique cultural and religious landscape [38] might provide insight into the adverse impact of negative life events on mental health, as these cultural and spiritual elements may act as protective factors, fostering resilience and coping mechanisms. Depression manifests differently across cultures [39]. Studies suggest cultures emphasising interdependence [40] and religiosity [38] may lower depression risk. Cultural norms also influence how depression is expressed and diagnosed [41,42]. These factors highlight the need for culturally sensitive approaches to understanding and addressing depression in Indonesia.

While numerous studies have explored negative life events and depression globally [19,20,43,44], research in Indonesia remains limited. Addressing these gaps, this study aims to examine the association between negative life events and depression among individuals in Indonesia, accounting for both individual and area-level factors. The findings of this study will provide evidence on how adverse experiences influence mental health and inform the development of preventive interventions.

## Methods

### Study population

Data come from the Indonesia Family Life Survey (IFLS-5) conducted in 2014−2015 by the RAND Corporation and Indonesian collaborators [45]. The IFLS was initiated in 1993 and included approximately 83% of Indonesia’s population, living in 13 of the 26 provinces. IFLS-5 provides individual data on depression, negative life events, health, demographics, and socioeconomic status [45].

To gain a more comprehensive understanding of the relationship between negative life events and depression in Indonesia, IFLS-5 data were enriched by linking them to district-level data sources from the Village Survey (Potensi Desa or Podes) and National Statistics in the same year. Podes data provides detailed infrastructure information reported by village heads, including the number of health facilities and physicians within each village. Aggregated data are then calculated for each district. Additionally, the IFLS-5 data were linked with Indonesia’s National Statistics data on the happiness index (measured by life satisfaction), population projections based on the 2010 census, and regional Gross Domestic Product (GDP) per capita. By combining these datasets, we capture the nested structure of individuals within districts. This enriched dataset strengthens the analysis by reducing measurement errors and enabling comprehensive adjustment for confounders at both the individual and district levels. We included a range of variables that may act as potential confounders influencing both exposure to negative life events and the risk of depression. These included individual-level factors (age, sex, marital status, education, employment status, per capita expenditure, functional status, body mass index, smoking status, and area of residence) and district-level characteristics (densities of healthcare providers and facilities, regional GDP per capita, and happiness index).

### Ethics approval

The IFLS surveys and their procedures were reviewed and approved by the Institutional Review Boards (IRBs) in the United States (at RAND) and in Indonesia (at the University of Gadjah Mada). Regarding participant consent, written informed consent was obtained from all respondents by the original data collection team before participation. For minors included in the survey, parental or guardian consent was obtained. The IFLS ensures ethical compliance by obtaining approval from recognised institutional review boards and adhering to international research ethics standards. Since this study involved secondary data analysis of de-identified data, no additional consent was required from participants.

### The dependent variable: depressive symptoms

Depression was measured using the validated 10-item Center for Epidemiologic Studies Depression Scale (CES-D-10) [46], which assessed symptoms experienced in the past week. Each item was rated on a 4-point Likert scale (0–3), with higher scores indicating greater depressive symptoms. CES-D-10 has shown good validity and reliability in the Indonesian population [47]. A score of ≥10 was used to categorise respondents as depressed, following established IFLS protocols [48,49].

### The main independent variable: negative life events

Negative life events were self-reported and aligned with prior research [29]**.** These included chronic illnesses (self or family member), natural disasters/accidents, and death of family members (spouse, parent, sibling, child, or relatives) (details in **S1 Table**). The number of events in the past year was categorised as 0, 1, or ≥2. A multimorbidity measure (presence of two or more chronic illnesses) was also constructed (analysis in **S2 Table**). We classified respondents as having multimorbidity if they reported being diagnosed with two or more chronic diseases, consistent with standard definitions used in previous studies to capture the cumulative burden of multiple conditions [50].

### Covariates

Following established practices, we controlled for potential confounding variables at the individual and district levels [51,52]. Individual-level covariates included demographics (age, sex), marital status (single as reference), education (primary school and lower as reference), and employment (unemployed as reference). We included the per capita expenditure variable as a log-transformed continuous variable [53]. District-level covariates included healthcare provider density, happiness index, and regional GDP. Per capita expenditure was chosen over income as an economic measure, aligning with practices in LMICs [54,55].

Functional status was measured using mobility, Activities of Daily Living (ADL) and Instrumental Activity of Daily Living (IADL). Functional status variables were included as continuous variables. Body mass index (BMI) was calculated by dividing weight in kilograms by height in meters squared and classified according to the Asia-Pacific guidelines as: underweight (<18.5 kg/m²), normal (18.5–22.9 kg/m², reference), overweight (23.0–24.9 kg/m²), and obese (≥25.0 kg/m²) [56]. We categorised smoking status into non-smoker (reference), past smoker and current smoker. Residential location was categorised into rural (reference) and urban areas.

We include the densities of healthcare facilities, which represent service availability at the district level. The densities of hospitals, primary care centres (*Pusat Kesehatan Masyarakat* or Puskesmas), integrated service posts (*Pos Pelayanan Terpadu* or Posyandu), medical doctors, and other health practitioners are used to measure the availability of healthcare providers. We further included the regional GDP per capita at the district level and the Happiness Index at the provincial level as the covariates.

### Statistical analysis

Descriptive statistics (mean, SD for continuous variables; frequency, percentage for categorical variables) summarised the data**.** Multilevel logistic regression models examined the association between negative life events (past 12 months) and depression, accounting for the nested structure (individuals within districts). We ran nested models: 1) simple adjusted models, which were adjusted for age and sex; 2) fully adjusted models, which were adjusted for individual (age, sex, employment status, education, marital status, per capita expenditure in quintiles, mobility, ADL, IADL, BMI, smoking behaviour and area of living) and district-level covariates (hospital, Puskesmas, Posyandu, doctor and other healthcare professionals densities, happiness index, and regional GDP/capita). We performed these models for two sets of independent variables: the number of negative life events and specific event types, such as chronic illness (self/family), natural disaster or accident, and death of a family member. Statistical analyses were performed using Stata 18.

## Results

Table 1 summarises the demographic, socioeconomic, and health-related characteristics of respondents by depression status. Among 31,446 participants aged 15 years and older, the overall prevalence of depression was 23.2%. Depression was more common among females (24.0%), younger individuals, and those reporting one (25.2%) or two or more (27.6%) negative life events compared with those who experienced none (22.1%). Among specific event types, depression was most prevalent among those exposed to natural disasters or accidents (29.8%) and those reporting chronic illness in themselves or family members (26.1%).

**Table 1:** Descriptive statistics on individual characteristics.

Variable	Total (N = 31,446)	Non-depressed (N = 24,134) (76.75%)	Depressed (N = 7,312) (23.25%)	Missing data (%)
Negative life events, n (%)				0
0	20,940 (66.59)	16,306 (67.56)	4,634 (22.13)	
1	9,566 (30.42)	7,148 (74.72)	2,418 (25.28)	
≥2	940 (2.99)	680 (72.34)	260 (27.66)	
Presence of chronic diseases (self or family member), n (%)				
Yes	8,262 (26.27)	6,109 (73.94)	2,153 (26.06)	0
No	23,184 (73.73)	18,025 (77.75)	5,159 (22.25)	
Natural disaster or accident, n (%)				
Yes	554 (1.76)	389 (70.22)	165 (29.78)	0
No	30,892 (98.24)	23,745 (78.86)	7,147 (23.14)	
The death of family members (spouse, parent, sibling, child, or relatives), n (%)				
Yes	2,649 (8.42)	2,025 (76.44)	624 (23.56)	0
No	28,797 (91.58)	22,109 (76.78)	6,688 (23.22)	
Age, mean (SD)	37.32 (14.93)	38.16 (15.03)	34.56 (14.24)	0.03
Sex, n (%)				
Male	14,700 (46.75)	11,403 (77.57)	3,297 (22.43)	
Female	16,738 (53.23)	12,725 (76.02)	4,013 (23.98)	0.03
Employed, n (%)				
Yes	21,031 (66.88)	16,220 (77.12)	4,811 (22.88)	0.01
No	10,413 (33.11)	7,913 (75.99)	2,500 (24.01)	
Education, n (%)				3.89
Primary and lower	9,291 (29.55)	7,099 (76.41)	2,192 (23.59)	
Secondary school	16,578 (52.72)	12,612 (76.08)	3,966 (23.92)	
College or higher	4,355 (13.85)	3,433 (78.83)	922 (21.17)	
Marital status, n (%)				0.03
Single	6,227 (19.8)	4,310 (69.21)	1,917 (30.79)	
Married	22,800 (72.51)	17,966 (78.8)	4,834 (21.2)	
Separated	804 (2.56)	566 (70.4)	238 (29.6)	
Widower	1,607 (5.11)	1,286 (80.02)	321 (19.98)	
Per capita expenditure, n (%)				6.59
5th quintile (Highest)	5,910 (18.79)	4,608 (77.97)	1,302 (22.03)	
4th quintile	5,931 (18.86)	4,600 (77.56)	1,331 (22.44)	
3rd quintile	5,911 (18.8)	4,503 (76.18)	1,408 (23.82)	
2nd quintile	5,863 (18.64)	4,502 (76.79)	1,361 (23.21)	
1st quintile (Lowest)	5,760 (18.32)	4,300 (74.65)	1,460 (25.35)	
Mobility, mean (SD)	0.19 (0.64)	0.17 (0.61)	0.24 (0.73)	0
Activity of Daily Living, mean (SD)	0.01 (0.14)	0.01 (0.12)	0.02 (0.19)	0
Instrumental Activity of Daily Living, mean (SD)	0.20 (0.70)	0.18 (0.66)	0.27 (0.81)	0
Body Mass Index, n (%)				1.27
Normal	3,798 (12.08)	2,795 (73.59)	1,003 (26.41)	
Underweight	12,615 (40.12)	9,506 (75.35)	3,109 (24.65)	
Overweight	4,826 (15.35)	3,789 (78.51)	1,037 (21.49)	
Obese	9,807 (31.19)	7,756 (79.09)	2,051 (20.91)	
Smoking, n (%)				0
Non-smoker	20,173 (64.15)	15,522 (76.94)	4,651 (23.06)	
Past smoker	1,433 (4.56)	1,148 (80.11)	285 (19.89)	
Current smoker	9,840 (31.29)	7,464 (75.85)	2,376 (24.15)	
Living in an urban area	18,558 (59.02)	14,207 (76.55)	4,351 (23.45)	0

Table 2 presents the distribution of healthcare resources across 297 districts, representing about 72% of all districts in Indonesia. On average, districts had 0.25 hospitals and 1.27 primary health centres (Puskesmas) per 10,000 population. Notably, districts with higher hospital density tended to have a lower prevalence of depression (r = –0.06, p < 0.05), suggesting that better healthcare access may help mitigate the mental health burden associated with adverse life experiences.

**Table 2:** Distribution of healthcare resources in districts (N = 297).

Variable	Mean (Standard Deviation)	Correlation with the proportion of depression in each district
Hospital density (/10,000 population)	0.25 (0.26)	−0.059*
Puskesmas density (/10,000 population)	1.27 (0.89)	−0.008
Posyandu density (/10,000 population)	11.45 (4.17)	0.003
Doctor density (/10,000 population)	2.10 (1.59)	−0.008
Other health practitioner density (/10,000 population)	12.56 (7.33)	−0.000
Happiness Index	68.48 (1.00)	−0.001
Regional Gross Domestic Product (GDP) per capita	44,897.48 (70,516.05)	−0.000

Note: * p < 0.05; ** p < 0.01; *** p < 0.001.

Table 3 shows that negative life events were significantly associated with higher odds of depression. Compared with individuals who had not experienced any events, those reporting one event had 1.24 times higher odds of depression (95% CI: 1.17–1.31), and those with two or more events had 1.48 times higher odds (95% CI: 1.27–1.72) after adjustment for age and sex. These associations remained significant after full adjustment for all covariates, suggesting a cumulative effect of multiple negative life events on mental health.

**Table 3:** Multilevel logistic regression analysis on the association between negative life events and depression among Indonesians.

Variables	Simple adjusted models^1^	Fully adjusted estimates^2^
Negative life events, reference = 0		
1	1.243 (1.172;1.318)***	1.220 (1.146;1.300)***
≥2	1.484 (1.274;1.729)***	1.550 (1.315;1.826)***
Random effects		
Intraclass correlation	0.053	0.049
Median Odds Ratio	1.508	1.481
Model diagnostics		
Observations	31,438	27,899
Clusters (Districts)	297	289

Note: ^1^adjusted with age and sex; ^2^adjusted with age, sex, socioeconomic status, mobility, ADL, IADL, BMI, health behaviour, rurality, density of health professionals and facilities, happiness index and regional GDP per capita. Significance: * p < 0.05; ** p < 0.01; *** p < 0.001.

As shown in Table 4, specific types of events were also associated with increased depression risk. Having a chronic illness (self or family member) was significantly related to depression (OR = 1.25; 95% CI: 1.17–1.33), and the effect was stronger among individuals with multimorbidity (OR = 1.95; 95% CI: 1.61–2.36). Experiencing natural disasters or accidents was linked to higher odds of depression (OR = 1.40; 95% CI: 1.14–1.72), while experiencing the death of a family member showed a modest but statistically significant association (OR = 1.13; 95% CI: 1.00–1.26). Overall, these results demonstrate that both the number and type of adverse life events are important determinants of depression in the Indonesian population.

**Table 4:** Multilevel logistic regression analysis on the association between negative life event types and depression among Indonesians. The models were performed separately for each negative life event type.

Variables	Simple adjusted models^1^	Fully adjusted estimates^2^
Presence of chronic diseases (self or family member)	1.274 (1.200;1.354)***	1.250 (1.171;1.334)***
Natural disaster or accident	1.355 (1.116;1.645)**	1.406 (1.145;1.726)**
The death of family members (spouse, parent, sibling, child, or relatives)	1.111 (1.008;1.225)*	1.139 (1.027;1.264)*

Note: ^1^adjusted with age and sex; ^2^adjusted with age, sex, socioeconomic, mobility, ADL, IADL, BMI, health behaviour, rurality, density of health professionals and facilities, happiness index and regional GDP per capita; ***Significant<0.001; **Significant<0.01; *Significant<0.05.

## Discussion

The findings of this study shed light on the association between the number of negative life events and depression among individuals using large data in Indonesia. It indicates a noteworthy association between experiencing multiple adverse life events and an increased likelihood of depression. Specifically, individuals who encountered two or more events exhibited 1.48 times higher odds of experiencing depression, even after adjusting for demographic, socioeconomic, health behaviours and area characteristics. In comparison, those who experienced one event had 1.24 times higher odds. This finding underscores the cumulative impact of life stressors on mental health, as highlighted in a meta-analysis study [20]. This result is consistent with previous studies conducted in older people in China [29], Hong Kong [24], the Netherlands [23], and the US [25].

This study distinguishes between different types of negative life events, revealing varying degrees of association with depression. Notably, the diagnosis of chronic diseases, either personally or within the family, emerged as a significant factor linked to depression. Individuals facing such health challenges had 1.25 times higher odds of experiencing depression, reinforcing the intricate interplay between physical and mental well-being. This result aligns with findings from previous cross-cultural studies [6,57,58], highlighting the reciprocal relationship between depression and chronic physical illness [59]. The co-occurrence of depression with chronic diseases, particularly in older adults, underscores the need for integrated interventions [60,61]. While successful interventions for chronic medical illnesses at the community level have been implemented in Indonesia through the Integrated Non-Communicable Diseases Health Post (Posbindu-NCD), operated by community health workers (CHWs) or Kader [62], similar interventions for depression are currently lacking. The WHO recommends using low-intensity psychological interventions, such as those delivered by non-specialists, to address the mental health gap in low-resource countries [63].

In Indonesia, recent policy developments underscore the importance of integrating mental health within community and primary care systems. The new Health Law No. 17 of 2023 and Government Regulation No. 28 of 2024 both emphasise community-based mental health care and position Puskesmas (primary health centres) as the main providers of mental health promotion, prevention, and early intervention. Our study reinforces this national direction by highlighting how community-level approaches can address the mental health impact of negative life events. This alignment with current policy priorities strengthens the public health relevance of our findings and supports the expansion of accessible, community-driven mental health services across Indonesia.

Furthermore, the study highlights the impact of natural disasters or accidents on mental health. Respondents who experienced these events within the last 12 months exhibited 1.4 times higher odds of having depression in the fully adjusted model. Previous studies showed several mental health problems following disasters, including depression [64,65]. These mental health problems prevalent and persist for a long period, even after the disaster [66,67]. This finding underscores the lasting psychological effects of environmental disruptions and accidents, indicating a need for targeted culturally sensitive mental health interventions in the aftermath of such events, such as Trauma-focused psychotherapies, including Cognitive Processing Therapy and Prolonged Exposure Therapy [68].

In line with prior research [19], we found that the loss of family members, encompassing spouse, parent, sibling, child, or relatives, is associated with depression a 1.13 times greater likelihood of depression. This number can be attributed to mourning factors, such as sudden death, conflicted relationships, or witnessing the death of a loved one [69]. Cultural elements in Indonesia, such as spirituality, might act as protective factors against depression [38,70]. These findings prompt further investigation into cultural, social, or coping mechanisms that could alleviate the impact of profound losses on mental health within the Indonesian context. Developing cost-effective, culturally sensitive, and sustainable low-intensity psychological interventions for those at risk has the potential to significantly reduce the burden of depression by increasing access to mental health support within the communities over time.

This study has several limitations. First, as a cross-sectional survey, it cannot establish temporal ordering between the onset of depression and the occurrence of negative life events. For instance, we lacked measures of depressive symptoms before and after each event. Consequently, the direction of the relationship between mental health problems and negative life events remains unclear. Although growing evidence indicates that negative life events increase the risk of developing mental health problems [16], other studies found that depression could lead to self-induced negative life events [71], particularly those with a negative cognitive style [72]. Furthermore, pre-existing depressive and anxiety symptoms have been shown to predict the occurrence of negative life events [73].

Secondly, the negative life events data we used were based solely on the verbal responses of respondents. Their answers may thus have been influenced by recall bias. Future data collection is needed to improve the measurement of individual past experiences. Finally, the information on the mediating and protective factors explaining the mechanism of the link between negative life events and depression in our study is limited. Culture shapes the response to negative life events and the experience of depression. Research in Indonesia supports the hypothesis that culture and religion are potential buffers against depression [38,70], suggesting that strong spirituality may lower depression. This implies that cultural practices and beliefs might provide a sense of belonging, purpose, and support, helping individuals cope with stress. However, it is important to acknowledge that the mechanisms behind this protective effect are not fully understood. Further research is needed to understand how cultural practices and beliefs protect against depression. This knowledge could be used to develop culturally sensitive interventions.

## Conclusion

This study highlights the significant association between negative life events and depression in the Indonesian population, particularly the cumulative effects of multiple events and the heightened risks linked to chronic illness and natural disasters. These findings emphasise the need for a comprehensive approach to mental health care that integrates psychological support into both healthcare and community-based systems. Expanding access through primary care outreach, community health workers, and disaster response programmes may help reach individuals who do not regularly access formal health services. Future research should explore cultural and familial factors that shape coping and resilience to inform contextually relevant mental health strategies in Indonesia.

## Supporting information

S1 Table The list of questions on negative life events. (DOCX)

S2 Table Multilevel logistic regression analysis on the association between multimorbidity and depression among Indonesians. (DOCX)
