# Supplementary material for: Negative life events and the risk of depression: Findings from Indonesia Family Life Survey 2014/2015
Source: PLoS One. 2026 Jan 9;21(1):e0319137. doi: 10.1371/journal.pone.0319137 (PMC12788663; doi:10.1371/journal.pone.0319137)
Supplement: S1 Table — (DOCX) [file pone.0319137.s001.docx]

**S1 Table.** The list of questions on negative life events

| Presence of chronic diseases (self or family member) | |
| --- | --- |
| CD05. | Have a doctor/paramedic/nurse/midwife ever told you that you had [...]   1. Hypertension 2. Diabetes or high blood sugar 3. Tuberculosis (TBC) 4. Asthma 5. Other lung conditions 6. Heart attack, coronary heart disease, angina, or other heart problems 7. Liver 8. Stroke 9. Cancer or malignant tumor 10. Arthritis/rheumatism 11. High cholesterol (Total or LDL) 12. Prostate illness 13. Kidney disease (except for tumor or cancer) 14. Stomach or other digestive disease 15. Emotional, nervous, or psychiatric problems 16. Memory-related disease |
| CD07. | When was the condition [...] first diagnosed? |
| Natural disaster or accident | |
| NDTYPE. | Type of disaster? |
|  | *included flood, landslide/mudslide, mudflow, volcanic eruption, earthquake, tsunami, windstorm, forest fire, fire, and drought. |
| ND05. | When was the most severe [...] in the last 5 years occurred? |
| ND12. | Did any member of the household suffer serious injury or illness because of [...] |
| The death of family members (spouse, parent, sibling, child, or relatives) | |
| EF03. | When did [...] pass away? |
| BA06a. | 12 months ago was your father/mother still alive? |
| BA29b. | How many siblings died during the past 12 months and were non-householders at the time of their deaths? |
| BA83a. | Child [...] still alive? |
| HH member relationship | |
| AR02b. | Relationship to household head now. |
|  | *included household head, husband/wife, child (biological), child (non-biological), son/daughter-in-law, parents, parent-in-law, sibling, brother/sister-in-law, grandchild, grandparent, uncle/aunt, nephew/niece, cousin, and other family. |
| RELATPROX. | Relationship with the deceased. |
|  | *included spouse, child, parent, sibling, and in-law |
