# Supplementary material for: Negative life events and the risk of depression: Findings from Indonesia Family Life Survey 2014/2015
Source: PLoS One. 2026 Jan 9;21(1):e0319137. doi: 10.1371/journal.pone.0319137 (PMC12788663; doi:10.1371/journal.pone.0319137)
Supplement: S2 Table — (DOCX) [file pone.0319137.s002.docx]

**S2 Table Multilevel logistic regression analysis on the association between multimorbidity and depression among Indonesians.**

| **Variables** | **Simple adjusted models^1^** | **Fully adjusted estimates^2^** |
| --- | --- | --- |
| Presence of multimorbidity | 1.99 (1.68; 2.37) | 1.95 (1.61; 2.36) |

Note: ^1^adjusted with age and sex; ^2^adjusted with age, sex, socio-economic, mobility, ADL, IADL, BMI, health behaviour, rurality, density of health professionals and facilities, happiness index and regional GDP per capita; ***Significant<0.001.
